# Mechanism-Oriented Biomaterial Strategies for Bone Regeneration in BRONJ: From Pathological Barriers to Evidence-Matched Repair

**DOI:** 10.3390/biom16091259

**Published:** 2026-08-31

**Authors:** Aiming Jiang, Juntong Liao, Yinyin Shi, Wenyan Song, Sisi Luo, Longjiang Li, Zhuoyuan Zhang

**Affiliations:** 1 State Key Laboratory of Oral Diseases, West China School of Stomatology, Sichuan University, Chengdu 610041, China; jiangaiming@stu.scu.edu.cn (A.J.); liaojuntong6987@gmail.com (J.L.); shiyinyin@stu.scu.edu.cn (Y.S.); songwysyy@gmail.com (W.S.); luosisi@stu.scu.edu.cn (S.L.); 2 Department of Head and Neck Cancer Surgery, West China School of Stomatology, Sichuan University, Chengdu 610041, China

**Keywords:** bisphosphonate-related osteonecrosis of the jaw (BRONJ), bone regeneration, biomaterial, osteoinduction, angiogenesis, reactive oxygen species, mechanism

## Abstract

Bisphosphonate-related osteonecrosis of the jaw (BRONJ) remains a challenging complication of bisphosphonate therapy because jaw extraction sockets exposed to bisphosphonates represent impaired wound environments rather than ordinary bone defects. This narrative review summarizes clinical, cellular, animal, and biomaterial evidence on the mechanisms that limit BRONJ repair and discusses how these pathological barriers can inform local material design. Current evidence suggests that BRONJ repair is constrained by impaired osteoclast-mediated remodeling, osteocyte and osteoblast dysfunction, oxidative stress, unresolved inflammation, angiogenic insufficiency, microbial challenge, mucosal instability, and changes in bone material properties. Biomaterial strategies investigated to date include local delivery of regenerative factors, restoration of remodeling activity, extracellular vesicles, nucleic acid nanostructures, platelet-derived matrices, antibacterial and ion-releasing hydrogels, angiogenic or lymphangiogenic systems, and mechanically adaptive scaffolds. Most studies remain preclinical and are based on rodent extraction or mandibular defect models, and few establish a direct causal link between a specific material property and durable BRONJ resolution. Future materials should be judged not only by their ability to enhance bone formation, but also by whether they can re-establish a sealed, vascularized, immune-balanced, and remodeling-competent socket capable of sustained jawbone repair.

## 1. Introduction

Bisphosphonate-related osteonecrosis of the jaw (BRONJ), the bisphosphonate-associated form within the broader spectrum of medication-related osteonecrosis of the jaw (MRONJ), commonly develops following tooth extraction or other dentoalveolar procedures [1]. Bisphosphonates can remain within the skeleton for prolonged periods [2]. By binding to bone mineral and being internalized by osteoclasts during bone resorption, these agents exert persistent effects on osteoclast-mediated remodeling [3]. Available pathophysiological models further implicate osteocyte injury and impaired angiogenesis [3,4], together with immune dysregulation, mucosal barrier damage, and microbial and inflammatory challenge [4,5]. Through their combined effects on necrotic matrix clearance, soft-tissue closure, vascular ingrowth, and new bone formation, these processes can convert an extraction socket into a persistent repair-deficient wound rather than a conventional osseous defect [3,4,5].

These interacting barriers distinguish BRONJ from conventional bone defects and may limit the effectiveness of materials designed primarily to provide osteoconduction, deliver regenerative cues, or maintain defect space within a permissive healing environment. Accordingly, this review examines bone remodeling and cellular dysfunction, redox imbalance, immune dysregulation, angiogenic impairment, microbial and mucosal constraints, and the jaw-specific mechanical environment in BRONJ. It then evaluates how regenerative signaling, osteoimmune regulation, vascularized osteogenesis, remodeling support, antibacterial and mucosal protection, and mechanical adaptation can be incorporated into biomaterial design to address these interconnected repair barriers.

Previous reviews have comprehensively summarized the broader pathophysiology, clinical presentation, and management of MRONJ [3,4]. In contrast, the present review restricts its evidence synthesis to bisphosphonate-associated disease and examines the extraction socket as a persistent repair-deficient environment rather than a conventional bone defect. It integrates remodeling suppression, cellular injury, redox imbalance, immune and microbial dysregulation, mucosal failure, vascular impairment, and jaw-specific mechanical constraints into a mechanism-to-material design framework. The review further distinguishes prevention or early repair from treatment of established lesions and separates BRONJ-specific intervention evidence from design principles extrapolated from conventional bone-regeneration studies. This framework is intended to clarify not only which biomaterial platforms have been investigated, but also when, in which disease context, and against which repair barrier their evidence is applicable.

## 2. Literature Search Strategy and Evidence Synthesis

This narrative review used a structured literature search to identify recent studies of biomaterial- and bioengineered platform-based interventions for BRONJ. PubMed, Web of Science Core Collection, and Scopus were searched for English-language articles published from 1 January 2021 to 31 May 2026. Disease-related terms, including “bisphosphonate-related osteonecrosis of the jaw,” “BRONJ,” “bisphosphonate-associated osteonecrosis of the jaw,” and combinations of “medication-related osteonecrosis of the jaw” with “bisphosphonate,” were combined with intervention-related terms such as “biomaterial,” “hydrogel,” “scaffold,” “membrane,” “carrier,” “nanoparticle,” “nanomaterial,” “drug delivery,” “platelet-rich fibrin,” “extracellular vesicle,” “exosome,” “nucleic acid nanostructure,” “bone graft,” and “bioactive glass.” Reference lists of eligible articles and relevant recent reviews were additionally screened to identify primary studies not captured by the database search. Original clinical, animal, and in vitro studies were included when they evaluated a biomaterial or bioengineered intervention for the prevention or repair of BRONJ, including hydrogels, scaffolds, membranes, grafts, delivery systems, nanoparticles or nanostructures, platelet-derived matrices, and extracellular-vesicle-based platforms. Mixed-exposure MRONJ studies were included only when bisphosphonate exposure or BRONJ-specific findings could be identified separately. Such studies were explicitly classified as mixed-exposure evidence rather than direct BRONJ evidence. Studies limited to non-bisphosphonate-related MRONJ, conventional bone regeneration without a BRONJ- or bisphosphonate-relevant model, purely mechanistic studies without a material-based intervention, reviews, conference abstracts, and protocols were excluded. The included studies were synthesized narratively and classified according to intervention context, including prevention at tooth extraction, treatment or reconstruction of established BRONJ, and in vitro feasibility. The material platform, experimental model, comparator, follow-up period, principal outcomes, limitations, and translational relevance were extracted. Evidence derived from non-BRONJ bone-regeneration studies was used only to inform proposed design directions and was not classified as BRONJ-specific intervention evidence. The final search was conducted on 31 May 2026. A total of 45 studies were retained, including two human clinical studies, 37 preclinical prevention or extraction/implantation repair studies, five preclinical studies addressing established lesions, and one BRONJ-relevant in vitro feasibility study.

Because the included studies encompassed heterogeneous clinical, animal, and in vitro designs, a single formal risk-of-bias tool was not applied across all studies. Instead, methodological strength and translational relevance were qualitatively appraised according to the relevance of the model to bisphosphonate-associated disease, comparator design, sample size, follow-up duration, intervention timing, completeness of BRONJ-specific outcomes, mechanistic validation, and clinical relevance. This appraisal informed the study-level assessment of methodological limitations and evidence categories, as well as the interpretation of each platform in the narrative synthesis. The appraisal was descriptive and was not intended as a formal grading of evidence certainty. Study selection, data extraction, and qualitative appraisal were independently checked by two authors, and disagreements were resolved through discussion.

## 3. Cellular and Molecular Mechanisms Underlying BRONJ

### 3.1. Suppression of Bone Remodeling and Cellular Coupling

Osteocyte injury and necrotic cell death serve as central events in bone remodeling disorders. In BRONJ, prolonged bisphosphonate deposition within jawbone remodels the osteocytic microenvironment in which necrotic cells can release damage-associated molecular patterns (DAMPs), amplify inflammatory signaling, disturb osteocyte network homeostasis, and propagate secondary injury [6]. Suppressed bone turnover further aggravates this process: altered remodeling in jaws exposed to bisphosphonates is associated with matrix necrosis, reduced mineral apposition, and detached or dysfunctional osteoclasts, indicating impaired coordination between osteoclasts and osteocytes [7,8]. Once osteocyte loss surpasses the compensatory capacity of the lacunocanalicular network, skeletal homeostasis becomes irreversibly impaired. At the molecular level, nitrogen-containing bisphosphonates inhibit farnesyl diphosphate synthase within the mevalonate pathway, depleting farnesyl pyrophosphate and geranylgeranyl pyrophosphate and impairing prenylation of small GTPases [9,10]. Beyond directly compromising osteoclast function, this mechanism may also affect osteoblast lineage cells, endothelial cells, and immune signaling. HMGB1/RAGE inhibition has been reported to reduce BRONJ-like lesion development in a zoledronate-treated mouse model, although downstream pathway interactions remain incompletely defined [11]. Therapeutic interpretation should therefore distinguish the established upstream drug action from downstream hypotheses that still require validation.

### 3.2. Osteoblast Dysfunction and Impaired Osteogenesis

At the lesion margin, defective new bone formation indicates that bisphosphonate exposure also compromises osteoblast lineage cells. Osteoblast dysfunction in BRONJ involves changes in osteogenic signaling, cell survival, matrix production, and coupling with other repair compartments. Previous studies have demonstrated that bisphosphonates can downregulate osteogenic genes such as OPN, OCN, and RUNX2 [12]. In human osteoblast-like cells, zoledronic acid (ZA) suppressed ALP, osteocalcin, RANKL, BSP, and COL1; some of these effects were partially rescued by enamel matrix derivative [13]. In MG63 cell cultures, Bisphosphonate administration also reduced secretion of osteoblast-derived factors, including BMP2, TGF-β1, IL-10, VEGF, and OPG [14]. At higher concentrations, ZA reduced viability, migration, ALP activity, and mineralized nodule formation in MC3T3-E1 cells and human osteoblast models. Histological specimens from BRONJ lesions further show necrotic trabeculae, absent osteoid deposition, and loss of osteoblast lining [15]. Since extraction socket healing depends on vascularized recruitment and survival of osteoprogenitors, angiogenic insufficiency may further reduce the available osteoblast precursor pool [4].

Osteogenic failure in BRONJ is best viewed as the combined outcome of direct toxicity to osteoblast lineage cells and indirect constraints imposed by vascular insufficiency, redox injury, chronic inflammation, and delayed matrix turnover.

### 3.3. Redox Imbalance and Oxidative Injury

Redox imbalance has been implicated in BRONJ-related tissue injury. Existing clinical and experimental evidence suggests that oxidative stress may contribute to cellular dysfunction, inflammatory amplification, endothelial impairment, and delayed socket healing, thereby interacting with remodeling suppression and vascular failure during disease progression. Clinically, patients with BRONJ have been reported to show higher markers of oxidative stress in serum and saliva, including malondialdehyde, oxidized glutathione, 8-oxo-7,8-dihydro-2-deoxyguanosine, and an increased GSSG/GSH ratio, compared with controls [16]. At the cellular level, ROS induced by bisphosphonate treatment inhibited proliferation and migration of oral fibroblasts [17]. In osteoclast precursors and mature osteoclast-like cells, apoptosis caused by ZA required ROS generation and was alleviated by either N-acetyl-L-cysteine treatment or p47phox knockdown [18]. Endothelial cell studies also indicate that nitrogen-containing bisphosphonates can reduce viability and induce oxidative and inflammatory metabolic adaptations [19].

These direct findings should be separated from broader redox literature. General bone biology supports the concept that physiological ROS participate in host defense and repair signaling, whereas persistent ROS excess can damage proteins and DNA, impair osteogenic differentiation, alter osteoclast activity, and destabilize vascular regeneration [20,21,22,23]. Studies of diabetic bone defects are useful only as comparators for compromised healing because they combine oxidative burden, reduced remodeling, angiogenic insufficiency, and chronic inflammation [24,25]. Together with direct evidence from BRONJ-related studies, findings from other compromised bone healing conditions support the possibility that ROS accumulation and oxidative injury are also involved in BRONJ lesions.

For biomaterial design, the most defensible interpretation is that redox imbalance modifies cell survival, inflammation, and vascular repair in BRONJ. It may become a therapeutic target when antioxidant, redox-modulating, or ROS-responsive systems are tested directly in models relevant to BRONJ; otherwise, ROS should be defined as a microenvironmental stressor that interacts with multiple pathogenic pathways, rather than an independent design mandate. Future studies need spatial and temporal evidence in human lesions and animal models, including markers such as 8-OHdG, lipid peroxidation, mitochondrial ROS, antioxidant response activation, and oxidative injury in defined cell populations.

### 3.4. Immune Dysregulation and Chronic Inflammation

BRONJ pathogenesis involves immune dysregulation in addition to suppressed bone remodeling [5]. Macrophages are especially relevant for the virtue of their multifaceted roles in host defense, debris clearance, repair signaling, and matrix remodeling [26,27,28,29]. The M1/M2 framework is simplified, but it remains useful for organizing inflammatory and reparative macrophage programs [30]. In BRONJ, bisphosphonate exposure, particularly ZA, is associated with increased M1 polarization, weaker reparative macrophage responses, and a higher M1/M2 ratio [31,32]. This imbalance may prolong inflammation beyond the physiological window required for wound repair.

The TLR4/NF-κB axis serves as a plausible link between oral infection, damaged bone, and macrophage-mediated inflammation in BRONJ [31,33]. Odontogenic infection and Gram-negative colonization provide lipopolysaccharide as a pathogen-associated molecular pattern, whereas necrotic bone and damaged cells release DAMPs [31]. These signals activate TLR4 on macrophages and initiate MyD88-dependent signaling, IκB degradation, NF-κB nuclear translocation, and transcription of inflammatory genes [34,35]. ZA has been shown to increase TLR4-mediated M1 macrophage polarization, and extraction models also show increased inflammatory cytokines after ZA administration [31,36].

Macrophage-centered descriptions do not fully capture the immune disturbance associated with BRONJ. In a mouse model combining zoledronic acid and dexamethasone, BRONJ-like lesions were associated with suppression of regulatory T-cell activity and expansion of IL-17-producing T-helper cells [37]. Single-cell analysis of oral tissues from zoledronate-treated mice further identified an altered T-cell compartment containing Cd8a-positive cytotoxic T cells and increased expression of the costimulatory molecule CD27 [38]. Neutrophil-mediated antimicrobial defense may also be compromised, as nitrogen-containing bisphosphonates impaired neutrophil chemotaxis, NADPH oxidase activity, and survival in experimental systems [39]. Consistent with this possibility, human BRONJ specimens showed reduced myeloperoxidase levels and downregulation of antibacterial-response genes despite increased expression of inflammatory mediators [40]. NLRP3/caspase-1-dependent IL-1β signaling provides an additional connection between danger sensing and persistent inflammation, although direct causal evidence is currently strongest in a diabetic BRONJ mouse model [41]. These observations extend BRONJ-associated immune dysfunction beyond macrophage polarization and suggest the coexistence of defective antimicrobial defense and failure of inflammatory resolution.

In this setting, cytokines and danger signals released from damaged cells and necrotic bone may reinforce NF-κB and inflammasome activity, prolonging inflammation beyond the physiological phase required for socket healing. The resulting microenvironment can compromise bacterial clearance, endothelial survival, osteoblast differentiation, and extracellular matrix deposition. Immune dysregulation therefore connects suppressed remodeling with microbial persistence and failure of vascular and osteogenic repair, although the relative contribution of individual immune populations is likely to vary with disease stage and experimental context.

### 3.5. Microbial Challenge and Mucosal Barrier Failure

The oral microbiota should not be treated as a uniformly pathogenic burden in BRONJ. Human BRONJ specimens contain polymicrobial communities and biofilms on necrotic bone, together with altered bacterial profiles and deficiencies in local antibacterial-response markers [40]. These findings support a close association between microbial colonization and poorly resolving lesions, but colonization of an already necrotic surface does not by itself demonstrate that bacteria initiate osteonecrosis. This distinction is supported by a zoledronate-treated mouse model in which prolonged depletion of indigenous microbiota exacerbated osteonecrosis following periapical inflammation and tooth extraction, suggesting that commensal microbial communities may retain protective functions [42]. The relevant disturbance is therefore better understood as a loss of host–microbiota balance involving pathogenic biofilm, pre-existing dental infection, and impaired host defense, rather than simply an increase in total bacterial burden.

Mucosal injury provides the anatomical interface through which this imbalance can sustain bone exposure. Nitrogen-containing bisphosphonates inhibit the proliferation and migration of oral epithelial cells and fibroblasts in vitro [17,43], while zoledronate-based extraction models show delayed gingival closure and deterioration of soft-tissue healing [44,45]. Incomplete re-epithelialization leaves the socket accessible to saliva, food debris, and microbial biofilms; continued microbial and inflammatory stimulation may, in turn, further impair epithelial migration, fibroblast-mediated matrix deposition, and connective-tissue organization. Microbial challenge and mucosal failure should therefore be viewed as mutually reinforcing barriers rather than as isolated consequences of infection or soft-tissue toxicity. For biomaterial design, this relationship indicates that antimicrobial performance should be evaluated together with epithelial closure, connective-tissue integration, microbial composition, and preservation of the host response required for wound resolution, rather than by planktonic bacterial killing alone.

### 3.6. Angiogenic Failure and Vascular Impairment

Angiogenic failure is increasingly recognized as a key component of BRONJ pathogenesis. Successful extraction socket healing requires timely vascular ingrowth to provide oxygen, nutrients, immune cell trafficking, and signals that support osteoprogenitors [46]. In BRONJ, this reparative vascular response is compromised. Animal studies have shown reduced vascular area in alveolar bone after ZA exposure and deterioration of hard and soft tissue healing in extraction sockets [44,47]. VEGF-related signaling is also relevant, although available evidence links it more directly to osteoclast differentiation or local VEGF delivery than to a fully defined human BRONJ pathway [48,49].

Endothelial dysfunction further limits vascular regeneration in BRONJ lesions. ZA has been reported to exert antiangiogenic effects on HUVECs, and alendronate or zoledronate can induce oxidative and metabolic adaptations in endothelial cells [50]. Bisphosphonate exposure is unlikely to act alone; inflammatory signaling and oxidative injury can further destabilize endothelial cells and convert the wound bed after extraction into a hypovascular and poorly regenerative niche. Vascular impairment in BRONJ also involves disruption of vascular niches associated with osteogenesis. Type H vessels, defined by high CD31 and endomucin expression, are closely associated with active bone formation and support osteoprogenitor recruitment, matrix deposition, and new bone formation [51]. In alveolar bone, ZA can reduce vascular area [47], and a magnesium nanocomposite hydrogel study in BRONJ models showed that repair was accompanied by type H vessel formation and Osterix-positive osteoprogenitor activation [52]. These findings suggest that vascular repair should be assessed by more than vessel density alone; stable, perfused networks that support osteogenesis are the clinically relevant target.

## 4. Biomechanical and Microenvironmental Constraints for Biomaterial Design in BRONJ

The pathological changes described above should not be treated as purely biochemical abnormalities. BRONJ develops within a mechanically loaded, microbially exposed, and inflammatory extraction socket. Jawbone is continuously subjected to masticatory loading, force buffering by the periodontal ligament, and dynamic alveolar remodeling [53]. Physiological mechanical forces help maintain periodontal and alveolar bone homeostasis [54], whereas excessive or dysregulated stimulation can impair osteocyte viability, disturb mechanosensing, and weaken local defense in already compromised bone [2]. Biomaterials for BRONJ should combine regenerative signaling with adaptation to the mechanical environment of the jaw. Tetradis et al. emphasized that bisphosphonates bind to mineralized bone and are internalized by osteoclasts during resorption, leading to osteoclast dysfunction and apoptosis [3]. After extraction, this becomes particularly detrimental because socket contouring and necrotic matrix clearance require remodeling mediated by osteoclasts. Prolonged bisphosphonate exposure has also been associated with microdamage accumulation and reduced trabecular mechanical competence [55,56]. Together, these findings support a design focus on both biological repair and mechanical compatibility.

### 4.1. Jaw-Specific Mechanical Context

Alveolar bone is not a mechanically inert structure; it belongs to a tooth, periodontal ligament (PDL), and alveolar bone unit that experiences cyclic masticatory loading. Functional occlusal loading helps maintain alveolar bone homeostasis, whereas occlusal unloading or hypofunction can induce unbalanced remodeling and net alveolar bone loss, a condition often described as alveolar disuse osteoporosis [57]. The PDL acts as a viscoelastic and mechanosensitive interface that absorbs, buffers, and redistributes occlusal forces to surrounding alveolar bone [58]. Within this unit, mechanical signals influence osteogenic, osteoclastic, vascular, and extracellular matrix responses, indicating that jawbone regeneration occurs in a cyclically loaded niche rather than a neutral defect environment [55]. At the cellular level, cyclic tensile stress that mimics physiological occlusal force increases SPP1, RUNX2, SP7, p-ERK1/2, and p-Elk1 in human PDL cells; blockade by U0126 implicates the ERK1/2-Elk1 MAPK pathway [59]. Cyclic tensile force can also suppress the osteoclastogenesis-supporting activity of PDL cells by inducing OPG through TGF-β signaling [60]. These benefits depend on force magnitude and context. Excessive cyclic compression, particularly under inflammatory conditions, increases TNF-α, IL-1β, IL-6, IL-8, MCP-1, RANKL, M-CSF, PTHLH, and CTSK, whereas moderate compression supports osteogenic gene expression [61]. Dysregulated loading may therefore shift periodontal mechanotransduction from homeostasis toward inflammation and resorptive signaling.

Tooth extraction disrupts the tooth, PDL, and alveolar bone unit and exposes the socket to oral micromotion, food impaction, salivary contamination, microbial challenge, and soft tissue tension [46]. In healthy sockets, coordinated angiogenesis, immune clearance, remodeling mediated by osteoclasts, epithelial migration, and new bone formation offset these stresses. In bone exposed to bisphosphonates, suppressed osteoclast activity may delay necrotic bone clearance, socket contouring, and microdamage repair. Experimental studies show that bisphosphonate administration before extraction delays early socket healing in rats [62], while ZA deteriorates hard and soft tissue healing in murine extraction sockets [44]. Lesions resembling ONJ induced by bisphosphonate or anti-RANKL treatment are also associated with impaired bone resorption and defective woven bone formation [63]. Site-specific effects of zoledronate on remodeling and mesenchymal osteogenic activity further support the idea that jaw repair depends on local biological context [64]. This mechanical vulnerability indicates that BRONJ biomaterials should move beyond biochemical delivery alone and incorporate matrices capable of adapting to the physical demands of the extraction socket. An ideal material for BRONJ should conformally fill irregular sockets, stabilize the early wound matrix, resist displacement under oral micromotion, and provide viscoelastic buffering without excessive stiffness. Its degradation kinetics should also match the delayed healing of bisphosphonate-exposed bone, maintaining temporary support while allowing vascular invasion, cell infiltration, and gradual remodeling.

### 4.2. Bisphosphonate-Associated Changes in Bone Material Properties

Conventional materials for bone repair are usually designed to provide osteoconduction, osteoinduction, and space maintenance for vascular invasion and new bone formation. Common options include autologous bone grafts, allografts, xenografts, demineralized bone matrix, calcium phosphate ceramics such as hydroxyapatite and β-tricalcium phosphate, bioactive glass, biodegradable polymer scaffolds, composite matrices, and guided bone regeneration membranes. Their efficacy generally depends on a functional host remodeling compartment in which scaffold integration, matrix turnover, vascular ingrowth, and bone deposition proceed together. This assumption is weakened in sockets susceptible to BRONJ, where bisphosphonate therapy suppresses remodeling mediated by osteoclasts and residual bisphosphonate on mineralized surfaces may remain biologically active during later repair attempts [65]. Material failure in this context may therefore reflect not only insufficient osteogenic or angiogenic stimulation, but also deterioration of the host bone substrate, including brittleness, reduced toughness, microcrack accumulation, and collagen matrix alteration.

Prolonged remodeling suppression permits fatigue microdamage to persist within mineralized bone [66]. Under physiological conditions, linear microcracks activate targeted remodeling: osteoclasts remove damaged matrix and osteoblasts restore tissue integrity. Chronic osteoclast inhibition uncouples microdamage generation from microdamage removal. In human trabecular bone, synchrotron X-ray micro-CT revealed that patients treated with bisphosphonates had fewer perforations but more numerous and larger microcracks than controls with or without fracture, accompanied by reduced tensile strength and Young’s modulus [55]. Another study reported 28% lower compressive strength than untreated fracture bone and 48% lower strength than bone from controls without fracture, with microcrack density increased by 24% and 51%, respectively [56]. Studies of extended bisphosphonate exposure further suggest that toughness-related properties are particularly vulnerable, making bone less able to absorb energy before fracture [67]. This distinction matters in extraction sockets, where repair requires active removal and replacement of damaged bone under repeated oral loading.

Drug-related changes in bone material properties extend beyond microcracks. Bisphosphonate therapy may preserve bone mass by suppressing resorption, but prolonged tissue residence time promotes secondary mineralization of older bone packets [68,69]. Increased mineralization can improve stiffness in some contexts, yet excessive mineral maturation may reduce ductility and impair energy dissipation during crack initiation and propagation [67]. FTIR analyses in canine bone showed that alendronate and risedronate increased mineral content and collagen maturity while narrowing mineral and matrix heterogeneity, a pattern consistent with increased brittleness and microcrack accumulation [70].

The organic matrix provides a complementary explanation for reduced toughness. Type I collagen contributes to energy dissipation and deformation after yield, and its mechanical performance depends on fibrillar organization and collagen crosslinks [71]. Remodeling suppression increases tissue age and promotes collagen maturation as well as accumulation of advanced glycation end product crosslinks such as pentosidine [72]. These changes can stiffen the matrix and reduce energy dissipation despite preserved bone volume [73].

Host bone deterioration during prolonged bisphosphonate therapy is therefore a multiscale process. Osteoclast inhibition limits targeted repair of damaged bone, while microcracks, increased mineralization, and altered collagen crosslinks jointly reduce toughness and renewal capacity. In sockets susceptible to BRONJ, this brittle and poorly renewable substrate may limit the efficacy of conventional bone substitutes unless materials also accommodate delayed remodeling and compromised bone quality.

## 5. Translational Implications for Biomaterial Design

**Figure 1:**
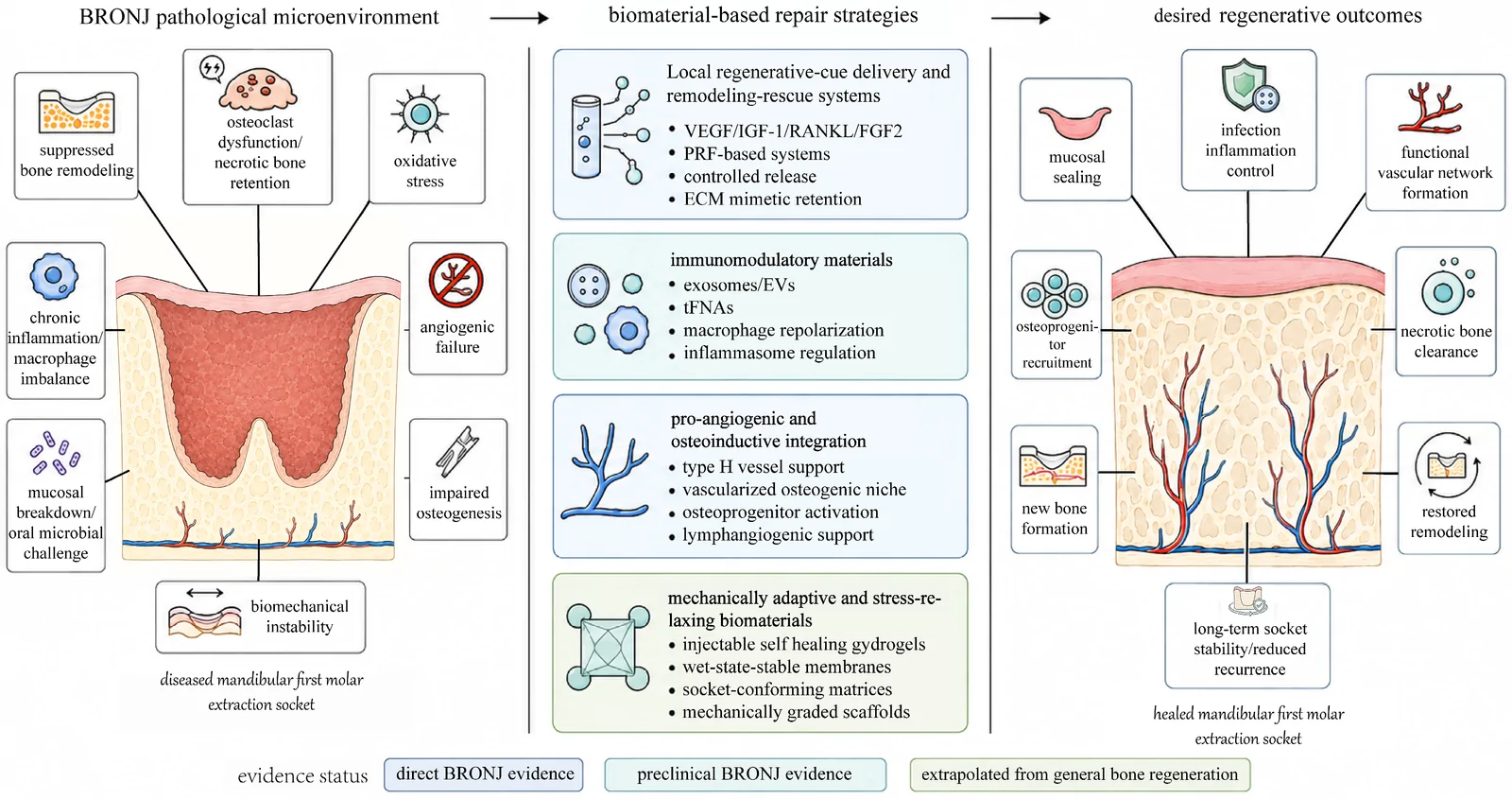
Mechanistic mapping of biomaterial strategies for bone regeneration.

Based on the disease barriers outlined above, biomaterial design for BRONJ should be framed as evidence-matched repair rather than as a simple extension of conventional bone regeneration. Figure 1 maps BRONJ-associated pathological barriers, including suppressed remodeling, persistent inflammation, angiogenic and osteogenic impairment, mucosal disruption, microbial challenge, and biomechanical instability, to corresponding material functions. Accordingly, the following sections synthesize current strategies according to their dominant design functions, including regenerative signaling, osteoimmune regulation, vascularized osteogenesis, remodeling support, and mechanical adaptation. Because many platforms exert overlapping effects, this functional organization is used to interpret how the interventions may address BRONJ-associated repair barriers, whereas Table 1 classifies the BRONJ-specific studies identified through the focused search according to intervention context and evidence stage.

BRONJ-associated repair barriers include suppressed bone remodeling, retention of necrotic bone, oxidative stress, persistent inflammation and immune dysregulation, mucosal breakdown and microbial challenge, angiogenic failure, impaired osteogenesis, and mechanical instability. Biomaterial strategies are mapped to these barriers according to their principal intended functions, while recognizing that individual platforms may exert overlapping effects. Evidence-status colors denote direct BRONJ evidence, preclinical BRONJ evidence, and evidence extrapolated from general bone-regeneration studies, as indicated in the figure legend. The other colors and labeled symbols are used only to visually distinguish pathological barriers, biomaterial strategies, and regenerative outcomes. The arrows indicate conceptual correspondence and the proposed progression from pathological barriers to repair strategies and desired outcomes, rather than uniformly established causal relationships or demonstrated clinical efficacy.

### 5.1. Local Delivery Systems for Regenerative Signaling

Local delivery of regenerative cues is one of the most direct material approaches for BRONJ prevention. Its rationale comes from BRONJ pathophysiology: bisphosphonate therapy suppresses bone remodeling, delays mucosal closure, alters angiogenesis, and creates a wound bed that is vulnerable to necrosis after dentoalveolar injury [3]. Local carriers may prolong factor retention within the socket and reduce systemic exposure, but the biological cue must match the stage and dominant bottleneck of repair.

In conventional bone repair, biomaterial carriers have been used to deliver osteogenic, angiogenic, chemotactic, and matrix remodeling factors, including BMP-2, BMP-7, VEGF, PDGF-BB, bFGF, TGF-β, IGF-1, and SDF-1 [74,75,76,77,78]. These factors are not interchangeable. BMPs promote osteogenic commitment and matrix mineralization; VEGF supports endothelial survival and angiogenesis; PDGF-BB recruits and expands mesenchymal and perivascular progenitors; bFGF contributes to fibroblast proliferation, angiogenesis, and early wound repair; and SDF-1 primarily supports progenitor recruitment [78]. Growth factor delivery should therefore be considered a way to reconstruct the temporal sequence of repair, not simply an osteoinductive supplement.

The broader bone regeneration literature shows that therapeutic efficacy depends less on selecting a universally superior molecule than on matching the delivered signal to the limiting step of the defect. BMP systems are usually aimed at osteogenic differentiation, VEGF or PDGF strategies at vascular and perivascular support, and chemokine systems at endogenous cell recruitment. When translated to BRONJ, the key question becomes whether the chosen cue can overcome the sequence of failure in a socket exposed to bisphosphonates. This concept has only been partially tested in material studies specific to BRONJ. A factor that enhances bone formation in a conventional defect cannot be assumed to work equally well in a socket affected by residual bisphosphonate, osteoclast suppression, soft tissue toxicity, oral contamination, and vascular insufficiency [79]. Delivery systems for BRONJ should therefore be evaluated with endpoints that reflect the disease phenotype, including mucosal coverage, exposed bone prevention, vascularized granulation tissue, necrotic matrix clearance, and durable socket remodeling, not osteogenic markers or mineral deposition alone.

Only a small subset of local biological cue studies directly addresses BRONJ. The bFGF-loaded gelatin hydrogel reported by Imada et al. is best interpreted as an early proof of concept. In a rat extraction model with ZA administration, gelatin hydrogel retained bFGF locally and was associated with improved mucosal coverage, increased new bone formation, and fewer changes resembling BRONJ compared with blank hydrogel or untreated controls [80]. This supports the value of local retention of a regenerative signal in a bisphosphonate-impaired wound, but it does not establish bFGF superiority over other factors or define bFGF materials as an established therapy.

Other cues address different pathological bottlenecks. Local RANKL delivery improved socket healing in bisphosphonate-treated rats, suggesting that partial restoration of osteoclast signaling can facilitate removal and replacement of damaged socket bone [81]. This approach targets remodeling suppression rather than classical osteoinduction. It also requires careful control because excessive or poorly localized osteoclast activation could aggravate bone loss, whereas insufficient activation may fail to clear necrotic matrix.

Platelet-derived matrices provide a broader but less controlled form of local cue delivery. PRP, PRF, L-PRF, and related autologous fibrin matrices release endogenous mixtures of PDGF, VEGF, TGF-β, FGF, and other cytokines, but they should not be equated with engineered single-factor systems [77]. In BRONJ management, their contribution is better described as wound support, clot stabilization, and assistance with soft tissue closure, especially when used with debridement or primary closure.

Overall, current evidence does not support presenting materials loaded with growth factors as an established therapeutic class for BRONJ. Local biological cue delivery remains a rational but underdeveloped translational direction. Existing studies point to several possible targets, including early mucosal repair, vascular support, progenitor recruitment, and remodeling rescue, but no single factor has emerged as a validated solution specific to BRONJ. Future platforms should aim to restore the ordered repair sequence of socket healing: soft tissue sealing, vascularized wound stabilization, controlled matrix turnover, osteoprogenitor recruitment, and new bone formation.

### 5.2. Immunomodulatory Materials

Recognition of immune dysfunction in BRONJ has shifted material design from passive defect filling toward active regulation of the local osteoimmune milieu. The goal is not broad immunosuppression. In extraction sockets exposed to oral microorganisms, excessive suppression may worsen infection, while unresolved inflammation perpetuates mucosal breakdown and necrotic bone exposure [5]. A useful immunomodulatory material should restrain destructive inflammation while preserving host defense, matrix turnover, and reparative cell recruitment.

Macrophage-directed modulation currently has the clearest rationale. Recent BRONJ studies show that ZA exposure is associated with early M1 macrophage predominance, increased inflammatory activity, and impaired collagen organization, whereas interventions that shift macrophage responses toward reparative phenotypes can reduce osteonecrosis and bone exposure in experimental models [32]. Materials that reprogram macrophage activity, attenuate the IL-1β/TNF-α axis, or limit inflammasome-related injury therefore fit the pathobiology of BRONJ.

Extracellular vesicle (EV) approaches provide direct cell-free evidence in BRONJ models. MSC-derived EVs prevented lesions resembling BRONJ in ZA-treated rats by reducing cellular senescence and senescence-associated inflammatory cytokines while promoting epithelial coverage, angiogenesis, and bone regeneration [82]. Adipose-derived mesenchymal stromal cell exosomes further refined this mechanism: ADSC exosomes accelerated gingival wound healing and socket bone repair, decreased IL-1β and TNF-α, and increased IL-1RA expression; IL-1RA-deficient exosomes partially lost this protective effect [83]. Another study linked ADSC exosome protection to inhibition of macrophage M1 polarization and pyroptosis through the NF-κB/NLRP3/IL-1β cascade [84]. EVs therefore appear to function as bioactive nanoscale mediators in BRONJ repair, but their translational value will depend on reproducible source selection, dose definition, cargo profiling, storage stability, and controlled delivery.

Nucleic acid nanostructures represent a distinct immunoregulatory platform. Tetrahedral framework nucleic acids have been reported to improve BRONJ repair by promoting angiogenesis, suppressing M1 polarization, and favoring M2 macrophage responses [85]. Because the nanostructure itself can be engineered as a programmable bioactive platform, this approach differs from protein delivery and cell-derived vesicles. The evidence remains preclinical, and future studies should clarify biodistribution, degradation, dose–response relationships, and whether macrophage polarization drives repair or reflects broader tissue recovery.

The magnesium nanocomposite hydrogel reported by Guo et al. is also relevant to immune regulation in BRONJ repair. In rat mandibular defects, this platform reduced local inflammatory burden and pathogen-associated stress, suggesting that control of inflammation and microbial challenge may help create a more permissive wound environment for tissue reconstruction [52].

Autologous platelet-derived matrices occupy an intermediate position between wound supporting materials and immune regulatory systems. PRF and related fibrin matrices contain leukocytes, platelets, fibrin, and endogenous cytokines, and may assist BRONJ management by stabilizing the clot, supporting soft tissue closure, and shaping the early inflammatory wound environment. A photocrosslinked Hep/GelMA hydrogel loaded with PRF controlled growth factor release, improved tissue reconstruction, upregulated osteogenic proteins, reduced inflammation, and maintained a more stable repair environment in a rat BRONJ model [86]. PRF systems should still be interpreted cautiously because their composition varies across patients and preparation protocols, and many studies assess healing outcomes without defining immune endpoints such as macrophage phenotype, inflammasome activation, neutrophil activity, or cytokine kinetics. Osteoimmune modulation may also be achieved by restoring signaling related to remodeling. A mesoporous silica nanoparticle hydrogel loaded with miR-21 improved local bone repair in a rat BRONJ model by restoring osteoclast differentiation and resorptive activity through the miR-21/PDCD4/NF-κB pathway, without evident systemic toxicity [87]. Although primarily remodeling-oriented, this strategy is relevant because osteoclasts and macrophages share monocyte lineage origins and inflammatory pathways. In contrast, topical active oxygen gel has only limited clinical evidence, mainly case observations, with proposed benefits involving oxygenation, antimicrobial activity, angiogenesis, collagen deposition, and wound healing [88].

Collectively, immunomodulation is a promising but still developing direction for BRONJ biomaterials. The strongest signals come from EV therapies, macrophage polarizing nanomaterials, and multifunctional hydrogels that integrate immune regulation with antibacterial and vascular regenerative effects. Future studies should move beyond general claims of inflammation control and define endpoints relevant to BRONJ, including M1/M2 dynamics, IL-1β/TNF-α/IL-10 balance, NLRP3 inflammasome activity, neutrophil and NET responses, microbial burden, mucosal closure, and necrotic bone clearance. The desired outcome is immune recalibration, not simple suppression.

### 5.3. Pro-Angiogenic and Osteoinductive Integration

Building on immune and inflammatory regulation, BRONJ-focused materials should also reconstruct a vascularized and osteopermissive repair niche. In sockets exposed to bisphosphonates, vascular impairment is coupled with poor progenitor recruitment, delayed matrix organization, suppressed turnover mediated by osteoclasts, and defective bone deposition [81]. Angiogenic design should therefore be judged by whether it supports osteogenesis and remodeling, not by endothelial markers or vessel density alone.

Localized angiogenic factor delivery has shown feasibility in models related to BRONJ. Sharma et al. reported that a gelatin and hyaluronic acid hydrogel carrying VEGF improved healing in ZA-treated extraction sockets, suggesting that local retention of angiogenic cues can partly counteract vascular suppression associated with bisphosphonate exposure [48]. This remains early proof-of-concept evidence. In BRONJ, vascular recovery must contribute to mucosal sealing, inflammatory resolution, osteoprogenitor recruitment, and later bone replacement; a short-lived angiogenic stimulus is unlikely to be sufficient by itself.

Evidence on type H vessels refines this concept. Type H vessels, characterized by high CD31 and endomucin expression, are closely associated with active osteogenesis and perivascular osteoprogenitor localization [51]. In alveolar bone, occlusal force-related homeostasis has been linked to type H angiogenesis, indicating that jawbone vascular repair is a specialized osteogenic niche response rather than nonspecific neovascularization [57]. This distinction is especially relevant to BRONJ, where new vessels must form within a mechanically loaded, contaminated, and remodeling-suppressed socket. The same magnesium nanocomposite hydrogel further illustrates how vascular and osteogenic repair can be coordinated in an impaired jawbone environment. Its regenerative effect was associated with type H vessel formation and Osterix-positive osteoprogenitor activation, indicating that reconstruction of a vascularized osteogenic niche may be more relevant to BRONJ repair than an increase in vessel density alone [52]. This moves the therapeutic focus from passive defect filling toward reconstruction of a functional vascularized regenerative niche. Its effects on inflammation and antimicrobial defense, discussed above, likely contribute to this integrated outcome.

The osteoinductive component should also include remodeling permissiveness. Local RANKL delivery partially restored osteoclast function and improved socket healing in animals treated with bisphosphonates [81]. Likewise, a mesoporous silica nanoparticle hydrogel loaded with miR-21 promoted BRONJ bone repair by restoring osteoclast differentiation and resorptive activity through the miR-21/PDCD4/NF-κB axis [87]. These strategies are not classical angiogenic therapies, but they matter for vascularized bone regeneration because vessels and osteogenic cells require a matrix that can be cleared, invaded, and replaced.

Biologic and nucleic acid systems further illustrate the interdependence of vascular repair, immune control, and stromal recovery. EV approaches are not reintroduced here as independent angiogenic therapies; instead, they show that endothelial recovery, inflammatory resolution, progenitor activity, and osteogenesis are coordinated during BRONJ repair. Similarly, tetrahedral framework nucleic acids, which promote angiogenesis while favoring reparative macrophage polarization, exemplify the overlap between vascular repair and immune recalibration [89].

Lymphangiogenic repair is an emerging extension of this concept. Cao et al. recently described an IGF-1-loaded hydrogel that promoted angiogenesis and lymphangiogenesis and prevented lesions resembling BRONJ in rats [90]. This direction remains less mature than angiogenic or osteogenic material design, but it is relevant to chronic oral wounds characterized by persistent inflammation, microbial stimulation, and impaired fluid clearance. Future studies should determine whether lymphatic reconstruction directly contributes to lesion resolution, mucosal closure, and bacterial clearance, or instead reflects secondary recovery after improved angiogenesis and osteogenesis.

In summary, angiogenic and osteoinductive integration is promising but still immature for BRONJ biomaterials. The most informative systems are those tested in models relevant to BRONJ and able to connect local signal retention, matrix stabilization, vascular niche reconstruction, osteoprogenitor activation, and remodeling rescue. Future studies should look beyond isolated markers such as VEGF, CD31, ALP, or OCN and test whether materials restore a coordinated repair sequence: perfused vascular networks, type H vessel recovery, perivascular osteoprogenitor recruitment, mucosal sealing, necrotic bone clearance, and prevention of recurrent bone exposure over time.

### 5.4. Mechanically Adaptive Design: Evidence Gaps and Extrapolated Principles

Once biological signaling, immune recalibration, and vascularized osteogenesis are considered, the physical behavior of the implanted material becomes critical. In sockets susceptible to BRONJ, the matrix is exposed to mucosal deformation, salivary hydration, disturbance from food, clot contraction, and occlusal micromotion before stable bone replacement occurs [54]. These stresses are difficult to tolerate in bisphosphonate-exposed bone, where remodeling is delayed and damaged matrix cannot be rapidly replaced [2]. The mechanical target is therefore not maximal stiffness, but controlled deformability, stability in the wet state, stress dissipation, and load transfer compatible with remodeling.

This view differs from scaffold selection based mainly on compressive modulus or space maintenance. In a mechanically active socket, an overly weak material may collapse or lose barrier function after hydration, whereas an excessively rigid one may concentrate interfacial strain, impair mucosal adaptation, and shield provisional tissue from physiological cues. Viscoelastic matrices with stress relaxation are attractive because they can dissipate deformation over time and permit cell-mediated matrix remodeling [91]. Direct BRONJ evidence is still lacking, so these mechanical concepts should be treated as design hypotheses requiring validation in oral wounds conditioned by bisphosphonate exposure, not as established therapeutic mechanisms.

Mechanics in the wet state are particularly important in oral wounds. Collagen membranes are widely used in guided bone regeneration, yet swelling, enzymatic degradation, and weak suture retention can compromise their function after exposure to biological fluids. Photoactive atelocollagen membranes show that crosslinking architecture can tune swelling ratio, compression modulus, proteolytic stability, and suture holding capacity [92]. Although these membranes were not tested in BRONJ, the design principle is relevant: primary closure, barrier stability, and resistance to early disruption are prerequisites for preventing recurrent bone exposure. Barrier materials define the mechanical boundary conditions for epithelial migration, vascular invasion, and bone deposition.

Dynamic hydrogels offer another route to mechanical adaptation. Reversible coordination bonds, dynamic covalent linkages, supramolecular interactions, and hybrid physical or chemical crosslinking can produce shear thinning, self-recovery, and stress relaxation [91]. These properties are attractive for irregular extraction sockets because an injectable material must conform during placement, recover cohesion after injection, and maintain contact with mucosa and bone under micromotion. Iron-coordinated hyaluronic acid hydrogels, for example, combine rapid gelation, shear tolerance, and self-healing behavior [93]. These are not BRONJ studies, but they identify material properties suited to the mechanical instability of oral wounds after extraction.

For bone repair, stress relaxation must be balanced with osteoconductive support. Soft hydrogels accommodate mucosal deformation but may not support mineralized tissue formation, while highly mineralized scaffolds support bone ingrowth but often lack compliance at the mucosal interface. Composite systems offer a rational compromise. GelMA and other light-based composite hydrogel platforms in the broader bone engineering literature show that cavity filling, degradability, mineral reinforcement, and mechanical tunability can be combined within cell-supportive matrices [94,95]. For BRONJ, the desirable design is not simply hard or soft, but a graded or multiphasic interface between mucosa, provisional matrix, and mineralizing bone.

Mineral scaffolds tested under compromised skeletal conditions further show why mechanics, vascular support, and remodeling permissiveness must be considered together. Mesoporous bioactive glass/polycaprolactone scaffolds promoted bone formation, vascularization, and osteoblast and osteoclast presence in osteoporotic sheep, whereas local zoledronate incorporation impaired osteoclast differentiation and bone healing despite the scaffold architecture [96]. Silicon-substituted hydroxyapatite scaffolds decorated with VEGF also supported vascularized bone regeneration in osteoporotic sheep, depending on scaffold microstructure and biological functionalization [97]. By analogy, an osteoconductive and structurally stable scaffold may still fail in BRONJ if it blocks osteoclast access, creates a persistent interface that cannot be remodeled, or does not permit vascularized matrix replacement.

Ligand presentation adds an interfacial level of mechanical control. Cell adhesion is a traction-bearing event through which cells sense matrix resistance and initiate osteogenic or angiogenic signaling. A biomaterial with dual integrin targeting ligands was recently shown to regulate endogenous cell adhesion and promote vascularized bone regeneration in adult and fetal bone defects [34]. In BRONJ, such strategies will be useful only if they are integrated into materials that tolerate oral disturbance and remain permissive to delayed remodeling. Mechanical adaptation should therefore be considered at three levels: bulk viscoelasticity for stress dissipation, interfacial mechanics for mucosal and cellular adhesion, and scaffold architecture for load transfer and remodeling access.

The biomaterial and bioengineered interventions identified through the focused literature search span clinical, animal, and in vitro settings. Although individual studies often emphasize a principal therapeutic objective, many platforms are designed to address multiple BRONJ-associated repair barriers, including impaired wound closure, persistent inflammation or infection, deficient angiogenesis and osteogenesis, and suppressed bone remodeling. Table 1 summarizes the BRONJ-specific studies retained from the 2021–2026 search and groups them according to intervention context and evidence stage: human clinical evidence, preclinical prevention or repair at extraction or implantation, preclinical treatment or reconstruction of established lesions, and BRONJ-relevant in vitro feasibility. This organization reflects when and in what model each intervention was evaluated rather than assigning multifunctional platforms to a single mechanistic category. Although it predates the 2021–2026 focused search window and is therefore not included in Table 1, the bFGF-loaded gelatin hydrogel reported by Imada et al. provides an early proof of concept for local regenerative-cue delivery in a bisphosphonate-treated extraction model.

**Table 1:** BRONJ-specific biomaterial and bioengineered interventions identified in the focused literature search (2021–2026).

Reference	Material	Model and BPs Exposure	Intervention Context	Main Outcomes	limitations	Evidence Category
**Human clinical evidence**						
Yüce et al., 2021 [98]	Autologous concentrated growth factor (CGF) matrix adjunctive to conservative surgery	Randomized study in osteoporotic women receiving oral bisphosphonates with established BRONJ	Treatment of established disease	CGF was associated with improved postoperative tissue healing indices, but between-group differences were not statistically significant.	Small single-center clinical study; early-stage disease and osteoporosis population; no definitive efficacy signal.	Human randomized controlled study
Ye et al., 2026 [99]	Autologous CGF placed in extraction sockets	119 patients involving 150 extraction sites with documented current or previous intravenous bisphosphonate exposure; a subset had also received concomitant or sequential denosumab.	Prevention at extraction	CGF reduced BRONJ incidence overall and improved early gingival healing/keratinized tissue measures.	Single-center trial; concomitant or sequential denosumab exposure in a subset limits attribution exclusively to bisphosphonate-associated disease.	Mixed-exposure human randomized controlled study
**Preclinical prevention or repair at extraction/implantation (including BP-impaired wound models)**						
Soundia et al., 2021 [81]	Recombinant RANKL delivered locally on a collagen carrier	Zoledronic acid-treated rat extraction sockets; split-site comparison	Prevention at extraction	Local RANKL increased osteoclast attachment and granulation/socket healing and reduced necrotic-bone retention despite continued ZA exposure.	Very short follow-up; pathway rescue may increase resorption if dose or release is not tightly controlled	Small-animal prevention model
Sharma et al., 2021 [48]	VEGF encapsulated in a gelatin-hyaluronic acid hydrogel	Zoledronic acid-treated rodent maxillary extraction model	Prevention at extraction	VEGF-hydrogel improved vascularized socket repair and reduced inflammation and osteonecrotic features relative to controls.	Single growth-factor dose and short rodent follow-up; local release kinetics and oncologic safety were not clinically addressed.	Small-animal prevention model
Tanaka et al., 2021 [100]	E. coli-derived rhBMP-2 adsorbed to beta-tricalcium phosphate (beta-TCP)	Cyclophosphamide + zoledronic acid BRONJ-like mouse extraction model	Prevention and early treatment	E-rhBMP-2/beta-TCP reduced necrotic bone; bone-density benefit was clearer when applied preventively.	Mouse model with chemotherapy co-exposure; efficacy depended on application timing; no long-term safety assessment.	Small-animal prevention/treatment model
Razmara et al., 2021 [101]	Collagen scaffold saturated with autologous platelet-rich plasma (PRP)	17 zoledronic acid-treated male Wistar rats with mandibular extraction sockets	Prevention at extraction	The PRP-collagen composite increased new bone/osteoblast indices and reduced inflammatory and necrotic changes.	Small groups and animal-only evidence; autologous PRP composition and preparation are variable.	Small-animal prevention model
Huang et al., 2021 [102]	Small extracellular vesicles derived from adipose tissue (sEV-AT)	Bisphosphonate-related rat extraction model	Prevention at extraction	sEV-AT increased local angiogenesis and soft-tissue/bone healing and reduced lesion extent.	Systemic/biologic EV product with unresolved potency, biodistribution, manufacturing and- dose-standardization issues.	Small-animal prevention model
Kushiro et al., 2021 [103]	Dental pulp stem cell-conditioned medium retained in atelocollagen or gelatin sponges	Zoledronic acid-treated rat extraction sockets	Prevention at extraction	Atelocollagen-supported conditioned medium reduced bone exposure/empty lacunae and increased VEGF-positive cells, consistent with sustained local signaling.	One-week endpoint; conditioned-medium composition and batch reproducibility were not standardized for translation.	Small-animal prevention model
da Silva et al., 2022 [104]	Inorganic bovine bone graft or beta-TCP socket graft	Zoledronic acid-treated Wistar rat extraction model	Prevention at extraction	beta-TCP increased bone formation and reduced empty lacunae, but grafted groups still displayed clinical BRONJ manifestations.	Mixed/partly discordant outcomes; graft retention and impaired remodeling may limit benefit in low-turnover bone.	Small-animal prevention model
Sarkarat et al., 2022 [105]	Hyaluronic acid (HA), absorbable collagen sponge (ACS), or HA + ACS	40 zoledronic acid-treated rats after first-molar extraction	Prevention at extraction	HA, alone or with ACS, improved histomorphometric socket healing and reduced BRONJ-like changes compared with untreated controls.	Preliminary animal study; no micro-CT or long-term lesion recurrence assessment; material dose/release was not optimized.	Small-animal prevention model
Zhao et al., 2022 [89]	Tetrahedral framework nucleic acid carrying the angiogenic peptide KLT (tFNA-KLT)	Zoledronic acid-treated rat extraction model plus BP-relevant cell assays	Prevention at extraction	tFNA-KLT promoted angiogenesis, favorable macrophage responses and extraction-socket repair, lowering BRONJ-like lesion burden.	Single rodent model; nucleic-acid stability, scale-up, off-target signaling and long-term safety remain unresolved.	Small-animal + in vitro prevention study
Okawa et al., 2022 [38]	Hydroxymethylene diphosphonate in deformable nanoscale lipid vesicles (HMDP-DNV)	Legacy nitrogen-containing BP-loaded mouse jaw with maxillary extraction	Prevention at extraction	Local HMDP-DNV displaced legacy N-BP from jawbone and improved gingival closure/socket healing while reducing osteonecrosis.	Mechanism and efficacy established in mice; formulation dosing, local pharmacokinetics and human mucosal safety need validation.	Small-animal prevention model
Zhu et al., 2022 [106]	Biodegradable magnesium implant	50 zoledronic acid/dexamethasone-treated rats with mandibular defects	Implant-associated prevention/repair	Magnesium reduced lesion frequency, increased new bone and VEGFA/CGRP-linked angiogenesis; pathway inhibition attenuated benefit.	Rigid implant model rather than extraction socket; corrosion, gas formation and dose control require translation to human defects.	Small-animal implant model
Hadad et al., 2022 [107]	beta-TCP alone or combined with antimicrobial photodynamic therapy or local doxycycline	72 zoledronic acid-treated Wistar rats after molar extraction	Prevention at extraction	beta-TCP-based protocols, particularly with antimicrobial adjuncts, improved alveolar repair and reduced BRONJ-like features.	Many small experimental groups; treatment effects cannot be attributed solely to the scaffold; no long-term follow-up.	Small-animal prevention model
Sacco et al., 2023 [108]	Doxycycline-loaded hydroxyapatite (HADOX)	35 female Wistar rats exposed to different zoledronic acid doses and dentoalveolar surgery	Risk reduction at surgery	HADOX increased new bone and reduced inflammation relative to carrier/control conditions.	Short rat study; residual material and antibiotic-resistance/local-dose questions limit direct translation.	Small-animal prevention model
Funayama et al., 2023 [109]	beta-TCP granules placed in extraction sockets	48 high-dose zoledronic acid-treated male Sprague-Dawley rats	Prevention at extraction	beta-TCP reduced local ZA deposition and necrotic bone and improved mucosal and osseous healing, with changes in autophagy-associated proteins.	High-dose rodent exposure and material-specific effects; mechanism of reduced local BP deposition requires further confirmation.	Small-animal prevention model
Zheng et al., 2023 [83]	Adipose-derived mesenchymal stromal cell exosomes enriched in IL-1RA activity	Zoledronate-associated rodent extraction model plus gingival cell studies	Prevention at extraction	Exosomes promoted primary gingival closure and reduced BRONJ-like lesions through IL-1RA-associated anti-inflammatory signaling.	EV identity/potency and dosing are not standardized; mechanistic rescue does not establish clinical manufacturing feasibility.	Small-animal + in vitro prevention study
Etemadi et al., 2023 [110]	Autologous leukocyte- and platelet-rich fibrin (L-PRF)	28 zoledronic acid-treated rats; bilateral maxillary extractions (split-mouth)	Prevention at extraction	L-PRF reduced histologic necrosis and inflammation and lowered the frequency of BRONJ-like changes	Split-mouth rodent design; platelet product variability and lack of blinded long-term outcomes limit certainty.	Small-animal prevention model
Zhang et al., 2023 [111]	Tetrahedral DNA nanomaterial carrying four TLR4-siRNAs (TDN-TLR4-4siR)	Zoledronic acid-primed macrophages and rat extraction model	Prevention at extraction	The platform restored macrophage mitochondrial homeostasis, shifted polarization away from M1, reduced sequestra and accelerated wound healing.	Complex siRNA nanomedicine; biodistribution, immunogenicity, manufacturing reproducibility and long-term safety are unresolved.	Small-animal + in vitro prevention study
de Sousa et al., 2024 [112]	1% Agaricus blazei polysaccharide (PAb) gel	54 female rats; zoledronic acid 0.1 mg/kg three times weekly with maxillary extraction	Prevention at extraction	PAb gel improved mucosal healing and osteocyte viability, reduced sequestration and enhanced Wnt/Runx2-linked bone remodeling and matrix quality.	Short rodent study of a complex natural product; composition, active constituents and batch consistency require definition.	Small-animal prevention model
Tao et al., 2024 [86]	Heparin/GelMA hydrogel loaded with platelet-rich fibrin (PRF)	48 female zoledronic acid/dexamethasone-treated rats after extraction	Prevention at extraction	The PRF-hydrogel composite improved mucosal/bone regeneration, reduced necrosis/inflammation and increased OCN, collagen I and BMP-2 expression.	Rodent-only study; PRF variability and photocrosslinked hydrogel manufacturing/sterility need clinical standardization.	Small-animal prevention model
Pellicano et al., 2024 [113]	F18 bioactive glass combined with a bovine pericardial membrane	26 zoledronic acid-treated male Wistar rats after extraction	Prevention at extraction	The composite reduced osteonecrosis, osteolysis and abscess formation while supporting tissue repair.	Small single-animal model; xenogeneic membrane safety and degradation and bioactive-glass dose remain untested clinically.	Small-animal prevention model
Cantoran-Castillo et al., 2024 [114]	Local polyguanidine conjugate (GuaDex)	Zoledronic acid-treated rats with mandibular osteotomy and Streptococcus gordonii challenge	Infection-focused prevention	GuaDex eliminated detectable bacteria, prevented ONJ-like lesions and supported vascularized bone regeneration.	Deliberate bacterial inoculation and short follow-up; local polymer toxicity and microbiome effects need broader testing.	Small-animal prevention model
Qin et al., 2024 [115]	Zoledronate/rapamycin-loaded nanoparticle (ZDPR)	Mouse BRONJ model and lymphatic endothelial cell studies, including conditional knockout models	Mechanism-linked prevention	ZDPR enhanced autophagy, reduced ER-stress apoptosis in lymphatic endothelial cells, restored lymphatic drainage and mitigated necrosis/inflammation.	Mechanistically intensive mouse work; a ZA-containing rescue formulation requires careful evaluation of local exposure and systemic benefit-risk.	Small-animal + in vitro prevention study
Dang et al., 2024 [116]	E-rhBMP-2/beta-TCP artificial bone graft	Zoledronic acid-associated BRONJ-like mouse extraction model	Prevention at extraction	The graft reduced osteocyte dendritic damage, microdamage and necrotic bone changes and improved socket repair.	Mouse extension of earlier BMP-2 work; ectopic bone, dose control and oncologic safety were not resolved.	Small-animal prevention model
Nishimaki et al., 2024 [117]	Allogeneic mesenchymal stromal cell sheet co-grafted with a titanium implant	24 zoledronic acid/dexamethasone-treated male rats	Implant-associated prevention	MSC sheets reduced necrotic bone and empty osteocyte lacunae, although bone volume fraction was not significantly improved.	Cell-sheet manufacturing and allogeneic-cell safety are complex; lesion reduction did not translate into a clear volumetric bone gain.	Small-animal bioengineered implant model
Zheng et al., 2024 [84]	Adipose-derived mesenchymal stromal cell exosomes	Zoledronate-associated rodent extraction model plus macrophage assays	Prevention at extraction	Exosomes inhibited M1 polarization and NLRP3-associated pyroptosis and improved soft-tissue and socket healing.	Potential overlap with the authors’ earlier EV program; product characterization and clinical-grade manufacturing remain limiting.	Small-animal + in vitro prevention study
Cao et al., 2025 [90]	IGF-1-loaded chitosan/gelatin/beta-glycerophosphate thermosensitive hydrogel	Zoledronic acid/dexamethasone-treated rat extraction model	Prevention at extraction	Sustained IGF-1 delivery restored PI3K/AKT signaling, angiogenesis and lymphangiogenesis and outperformed free IGF-1 injection in preventing lesions.	Single rodent model; growth-factor stability, dose, release and systemic/oncologic safety require validation.	Small-animal + in vitro prevention study
Li et al., 2025 [87]	miR-21-loaded mesoporous silica nanoparticle hydrogel	Zoledronic acid-associated rat extraction model plus osteoclast assays	Prevention/repair at extraction	The platform restored osteoclast differentiation/resorption through the miR-21/PDCD4/NF-kappaB axis and improved socket repair without evident short-term systemic toxicity.	Rodent-only study; miRNA off-target effects, silica clearance and long-term safety are unknown.	Small-animal + in vitro prevention study
Shi et al., 2025 [118]	Autologous PRP combined with Bio-Oss granules	18 rats receiving zoledronic acid 0.1 mg/kg three times weekly with mandibular extraction defects	Prevention at extraction	Bone exposure was absent in the composite group and micro-CT/histology showed higher BMD/BV/TV and fewer empty lacunae than controls.	*n* = 6 per group; Bio-Oss alone was ineffective; shared control data were later reused in a related 2026 study.	Small-animal prevention model
Kurokawa et al., 2025 [119]	0.3% bFGF mixed with 3% hydroxypropyl cellulose (HPC)	24 female rats given two intravenous ZA doses before extraction	Prevention at extraction	BRONJ incidence was 8.3% with bFGF-HPC versus 100% with HPC, accompanied by increased angiogenesis and new bone formation.	Small two-group study; large apparent effect needs independent replication and careful comparison with the earlier 2019 gelatin-hydrogel study.	Small-animal prevention model
Hadad et al., 2025 [120]	Locally applied 10% doxycycline gel	72 Wistar rats assigned to saline, ZA, or ZA + doxycycline gel protocols	Prevention at extraction	Local doxycycline improved mucosal/alveolar healing and reduced inflammatory and necrotic bone indices.	Short animal study; antimicrobial resistance, optimal local exposure and carrier contribution were not resolved.	Small-animal prevention model
Gachayev et al., 2025 [121]	Hyaluronic acid gel placed in the extraction socket	24 male Wistar rats; ZA 0.06 mg/kg weekly for 4 weeks	Prevention at extraction	HA restored multiple micro-CT parameters and histomorphometric bone formation toward control values and reduced empty lacunae.	Small study with one HA formulation/dose; no direct comparison with ACS or sustained-release formulations.	Small-animal prevention model
Hayashi et al., 2025 [122]	Copper-phosphate-modified carbonate-apatite honeycomb plug-in scaffold	Antibacterial/angiogenic cell assays and a bisphosphonate-associated extraction model	Prevention at extraction	The scaffold combined antibacterial, osteogenic and angiogenic functions, promoted bone/gingival closure and suppressed BRONJ-like lesions.	Complex inorganic scaffold with limited in vivo duration; copper release, degradation and defect-fit require translational optimization.	Small-animal + in vitro prevention study
Cui et al., 2026 [123]	Sericin-based multifunctional SHS@TCM nanoparticles	BRONJ-relevant macrophage/cell assays and rat prevention model	Prophylaxis of BRONJ	The nanoplatform reprogrammed macrophages, improved mitochondrial function and restored the inflammatory-regenerative microenvironment in the rat model.	Recently published single research program; detailed pharmacokinetics, component attribution, scale-up and long-term safety remain uncertain.	Small-animal + in vitro prevention study
Chen et al., 2026 [124]	Human umbilical-cord MSCs combined with Bio-Oss granules	24 rats; ZA 0.1 mg/kg three times weekly for 12 weeks; mandibular extraction defects	Prevention at extraction	The composite eliminated bone exposure in this small sample and improved mucosal coverage, BV/TV, BMD and osteocyte viability; Bio-Oss alone was ineffective.	*n* = 6/group; pairwise bone-exposure comparisons were not significant after correction; no in vivo cell tracking; controls shared with [118]	Small-animal cell-scaffold model
Tu et al., 2025 [125]	GelMA/polydopamine hydrogel carrying miR-218-modified alveolar mucosa-derived stem cells, with PTH	30 male rats; ZA/dexamethasone model with maxillary osseous-mucosal wounds created after drug exposure	Prevention/repair of a BP-impaired osseous-mucosal wound	The full combination was associated with faster soft-tissue closure, fewer sequestra, greater bone volume and recovery of osteoblast-osteoclast coupling.	Highly complex cell/gene/drug combination; contribution of each component and clinical manufacturing feasibility are uncertain.	Small-animal prevention/repair + in vitro study
Zhang et al., 2026 [126]	Bone-targeted zoledronate/rapamycin nanoparticles (ZDPR)	Macrophage mitophagy studies and zoledronate-based BRONJ models	Mechanism-linked prevention in a zoledronate-based BRONJ model	ZDPR restored macrophage autophagic/mitochondrial function and prevented BRONJ-like lesions in the reported model; benefits in osteoporosis and osteolysis were evaluated separately.	Related to the earlier ZDPR program; prevention in a small-animal BRONJ model and efficacy in other skeletal models do not establish treatment efficacy in established human BRONJ.	Small-animal + mechanistic prevention study
de Almeida Silva et al., 2026 [127]	Atorvastatin-loaded graphene oxide hydrogel	Female Wistar rats with a zoledronic acid-induced jaw-osteonecrosis model and tooth extraction	Prevention/repair at extraction	The atorvastatin hydrogel was associated with improved mucosal healing, osteocyte viability, bone-quality measures and angiogenesis, with reduced inflammation.	Published online in March 2026 but assigned to the August 2026 issue; one short-term animal model and no independent replication or clinical evidence.	Small-animal material prevention/repair study
**Preclinical treatment or reconstruction of established lesions**						
Jamalpour et al., 2022 [128]	A-PRF or L-PRF adjunctive to surgical resection, with or without photobiomodulation	Rat BRONJ lesions induced before treatment	Treatment of established disease	PRF after resection improved wound and bone healing; the PRF + PBM combinations produced the most favorable composite outcomes.	Multicomponent intervention and small groups prevent isolation of PRF effects; no clinical validation.	Small-animal treatment model
Guo et al., 2025 [52]	Injectable magnesium-oxide nanocomposite hydrogel crosslinked with NHS-functionalized hyperbranched PEG/protein	Debrided mandibular defects in BRONJ rats; separate mandibular-defect and iliac-crest-defect implantation studies in minipigs without an induced BRONJ model	Post-debridement reconstruction	Mg release coordinated antibacterial, immunomodulatory, angiogenic/type-H-vessel and osteogenic responses and enhanced mandibular regeneration.	Complex multicomponent mechanism; the minipig work supports implantation feasibility in ordinary bone defects, not large-animal BRONJ efficacy or human recurrence reduction.	Small-animal BRONJ treatment + non-BRONJ large-animal feasibility
Shuai et al., 2025 [129]	miR-145-3p-engineered MSC exosomes delivered in HyStem-HP hydrogel	Established BRONJ lesion model plus jawbone MSC ferroptosis assays	Local treatment of established disease	Local delivery suppressed IREB2-linked ferroptosis, restored osteogenic competence and improved micro-CT and new-bone outcomes.	Engineered EV + hydrogel product has substantial potency, cargo-control and manufacturing challenges; no large-animal/clinical data.	Small-animal treatment + in vitro study
Jamalpour et al., 2025 [130]	Piezosurgery with A-PRF or L-PRF, with or without photobiomodulation	49 rats with confirmed BRONJ signs 8 weeks after extraction	Treatment of established disease	PRF improved soft-tissue and bone healing; piezosurgery or PBM alone was insufficient, and combined PRF/PBM protocols performed best.	Multimodal design and seven small groups; no clear advantage of A-PRF over L-PRF; rodent-only evidence.	Small-animal treatment model
Sun et al., 2026 [131]	Autologous CGF combined with cortical bone perforation after debridement	26 female ZA-treated rats were reported; 2 were designated for CGF preparation and excluded from outcome procedures; established BRONJ preceded intervention	Treatment of established disease	CGF promoted repair; bone perforation improved early blood supply, and the combination produced the most complete epithelial and osseous healing at 4 weeks.	Small analyzed groups and 2- to 4-week follow-up; surgical and matrix effects cannot be fully separated; the accepted-manuscript reporting of animal allocation should be read carefully.	Small-animal treatment model
**BRONJ-relevant in vitro feasibility**						
Thavornyutikarn et al., 2023 [132]	Carboxymethyl chitosan hydrogel containing mesoporous silica nanoparticles co-delivering clindamycin and geranylgeraniol	Zoledronic acid-exposed osteoclast/osteoblast-lineage cultures with Streptococcus sanguinis challenge	In vitro feasibility	The dual system combined antibacterial activity with partial rescue of ZA-impaired bone-cell functions.	No BRONJ animal model; release, dosing and tissue compatibility remain feasibility-level evidence only.	BRONJ-relevant in vitro study

Direct evidence for stress-relaxing or mechanically adaptive biomaterials in BRONJ remains limited. This gap is important because BRONJ manifests in a mechanically active jaw environment, even though it is often studied as a biochemical or inflammatory wound healing disorder. Future materials should be evaluated not only by gelation time, compressive modulus, degradation, and histological bone formation, but also by swelling in the wet state, stress relaxation kinetics, resistance to cyclic deformation, suture retention, interfacial stability, mucosal dehiscence, and compatibility with remodeling mediated by osteoclasts. These parameters would link mechanical performance to clinically meaningful outcomes such as soft tissue sealing, prevention of recurrent bone exposure, vascularized matrix replacement, and durable socket remodeling.

## 6. Current Challenges and Controversies

Despite the growing number of biomaterial approaches proposed for BRONJ prevention and repair, several conceptual and translational controversies remain. The most fundamental issue is that many interventions designed to improve extraction socket healing are interpreted as therapies specific to BRONJ. A prophylactic material placed immediately after extraction in a bisphosphonate-treated animal does not necessarily predict performance in an established necrotic lesion after debridement. Preventive intervention, early wound stabilization, and reconstruction after osteonecrosis are different biological and surgical contexts, yet they are often grouped together [133,134]. A stricter evidence hierarchy is needed, distinguishing preventive socket models, treatment models with established necrosis, low-level clinical observations, and comparative clinical data.

Preclinical model heterogeneity constitutes another major limitation. Rodent extraction models reproduce delayed mucosal closure, necrotic bone persistence, inflammatory infiltration, and impaired remodeling, but they vary widely in animal species, drug type, cumulative dose, dosing interval, extraction site, use of dexamethasone or chemotherapy, infection induction, and histological lesion criteria [135]. Rodent jawbone also differs from human jawbone in remodeling dynamics, cortical architecture, occlusal loading, microbiota, and immune background. These differences are especially important for biomaterial studies because material performance depends on defect geometry, wound contamination, mucosal tension, and mechanical loading.

Outcome assessment remains inconsistent. New bone area, BV/TV, osteogenic marker expression, vessel density, and inflammatory cytokines are informative, but they do not fully capture successful BRONJ repair. A material may increase mineral deposition without durable mucosal sealing, or reduce inflammation without clearing necrotic bone. Conversely, clinical improvement may result from soft tissue stabilization rather than true regeneration of vascularized bone [136]. Serum bone turnover markers, radiographic scores, histological necrotic bone area, microbial burden, immune cell phenotyping, and mucosal healing rates have all been used, but none is universally accepted as a surrogate endpoint for BRONJ resolution [137].

Mechanistic attribution also requires caution. Hydrogels, membranes, platelet-derived matrices, EVs, and ion-releasing scaffolds often affect inflammation, angiogenesis, osteogenesis, antibacterial activity, and wound stabilization simultaneously, as illustrated by several multifunctional BRONJ material studies [132]. Improved bone formation after implantation may reflect mucosal closure, reduced infection, altered macrophage activity, vascular recovery, or simple wound protection, rather than direct osteoinduction. Similarly, reduced cytokine expression does not prove immune reprogramming unless immune populations, timing, and causal pathways are defined. This concern is especially clear for EV products, for which community guidance emphasizes source definition, vesicle characterization, potency testing, and transparent reporting [138,139]. Future studies should avoid assigning a dominant mechanism based only on downstream repair outcomes. Stronger claims require loss-of-function or blocking experiments, temporal analysis, and controls that separate matrix effects from bioactive cargo effects.

Clinical management controversies further complicate translation. The value of a bisphosphonate drug holiday remains uncertain because prolonged skeletal retention may limit the immediate benefit of treatment interruption [140]. Surgery is increasingly supported for advanced BRONJ, but conservative therapy remains appropriate for selected patients with limited symptoms, high surgical risk, or ongoing oncologic treatment [141,142]. Antibiotics, antimicrobial rinses, platelet concentrates, laser therapy, teriparatide, ozone or oxygen-based topical approaches, and primary closure have all been explored, but evidence remains heterogeneous and stage-dependent [143,144]. New materials should therefore be compared not only with untreated controls but also with clinically relevant standards such as debridement, primary closure, antimicrobial management, and established wound-supporting adjuncts.

Safety and manufacturability deserve equal attention. BRONJ often affects medically complex patients receiving bisphosphonates for malignancy, metastatic bone disease, or severe osteoporosis; some patients may also receive antiangiogenic or other anticancer therapies that could modify the clinical course. Local delivery of potent growth factors, nucleic acids, stem cell-derived vesicles, or immunomodulatory agents must therefore be assessed for oncologic safety, infection risk, ectopic tissue formation, immune perturbation, prolonged material persistence, and batch reproducibility [144,145]. A biologically powerful material is not necessarily more translatable than a simpler wound stabilizing scaffold if its safety profile, manufacturing consistency, and regulatory pathway remain unclear.

The central controversy is not whether local materials can improve healing in principle, but whether current evidence can identify which material property produces which clinically meaningful outcome. Most available studies establish feasibility rather than a validated therapeutic class. The field needs a continuous evidence chain from material property to microenvironmental modulation, from local tissue repair to durable mucosal coverage and bone remodeling, and ultimately from preclinical efficacy to reduced recurrence of bone exposure in patients.

## 7. Future Perspectives

Future BRONJ biomaterial research should move from general bone regeneration supplementation toward reconstruction of the extraction socket niche. BRONJ involves coupled failures in remodeling, mucosal healing, angiogenesis, immunity, microbial control, redox balance, and local mechanics. Material selection should be matched to clinical stage because prevention after tooth extraction and repair after established osteonecrosis are distinct surgical contexts. Preclinical studies need more standardized BRONJ models and clinically meaningful endpoints, including mucosal coverage, necrotic bone clearance, vascularized tissue replacement, and durable absence of bone exposure. Instead of maximizing a single function such as osteogenesis or angiogenesis, future platforms should coordinate wound stabilization, immune recalibration, vascularized osteogenesis, antibacterial protection, and degradation compatible with remodeling within the same local environment. Advanced systems such as EVs, nucleic acid nanostructures, responsive hydrogels, and mechanically adaptive scaffolds require rigorous characterization, potency testing, safety evaluation, and batch consistency before clinical translation. Ultimately, a successful biomaterial specific to BRONJ should be judged by whether it restores a stable, vascularized, immune-balanced, and remodeling-competent socket capable of sustaining jawbone repair over the long term.

## 8. Conclusions

BRONJ should be regarded as a context-dependent failure of bone regeneration resulting from coupled disturbances in bone remodeling, vascularization, immune–redox homeostasis, microbial control, and jaw-specific mechanics. Biomaterials may address these interacting barriers through localized delivery, osteoimmune and redox modulation, antimicrobial protection, promotion of vascularized osteogenesis, and mechanically adaptive wound support. However, current evidence is dominated by preclinical studies designed to prevent lesion development, whereas therapeutic efficacy in established BRONJ and direct human evidence remain limited; mixed-exposure clinical data also require cautious interpretation. Future studies should therefore use well-characterized bisphosphonate-specific models, distinguish prevention from treatment, evaluate clinically relevant healing and recurrence endpoints, and compare candidate materials with standard surgical or conservative management. Attention to biosafety, degradation, manufacturing reproducibility, and regulatory feasibility will also be essential. Overall, mechanism-oriented biomaterial design provides a promising framework for BRONJ repair, but clinical translation will depend on rigorous disease-specific validation rather than efficacy in conventional bone-defect models alone.

## Data Availability

The original contributions presented in this study are included in the article. Further inquiries can be directed to the corresponding authors.

## Abbreviations

8-OHdG, 8-hydroxy-2′-deoxyguanosine; A-PRF, advanced platelet-rich fibrin; ACS, absorbable collagen sponge; ADSC, adipose-derived mesenchymal stromal cell; ALP, alkaline phosphatase; bFGF, basic fibroblast growth factor; BMD, bone mineral density; BMP, bone morphogenetic protein; BP, bisphosphonate; BRONJ, bisphosphonate-related osteonecrosis of the jaw; BSP, bone sialoprotein; BV/TV, bone volume/total volume; CD31, cluster of differentiation 31; CGF, concentrated growth factor; CGRP, calcitonin gene-related peptide; COL1, type I collagen; CT, computed tomography; CTSK, cathepsin K; CuP, copper phosphate; DAMP, damage-associated molecular pattern; DNA, deoxyribonucleic acid; DNV, deformable nanoscale vesicle; ER, endoplasmic reticulum; ERK1/2, extracellular signal-regulated kinases 1 and 2; EV, extracellular vesicle; FGF, fibroblast growth factor; FTIR, Fourier-transform infrared spectroscopy; GelMA, gelatin methacryloyl; GSH, reduced glutathione; GSSG, oxidized glutathione; HA, hyaluronic acid; HADOX, doxycycline-loaded hydroxyapatite; Hep, heparin; HMDP, hydroxymethylene diphosphonate; HMGB1, high-mobility group box 1; HPC, hydroxypropyl cellulose; HPMC, hydroxypropyl methylcellulose; HUVEC, human umbilical vein endothelial cell; IGF-1, insulin-like growth factor 1; IL, interleukin; IL-1RA, interleukin-1 receptor antagonist; IREB2, iron-responsive element-binding protein 2; IκB, inhibitor of nuclear factor κB; L-PRF, leukocyte- and platelet-rich fibrin; M-CSF, macrophage colony-stimulating factor; MAPK, mitogen-activated protein kinase; MCP-1, monocyte chemoattractant protein 1; miR, microRNA; MRONJ, medication-related osteonecrosis of the jaw; MSC, mesenchymal stromal cell; MyD88, myeloid differentiation primary response 88; N-BP, nitrogen-containing bisphosphonate; NET, neutrophil extracellular trap; NF-κB, nuclear factor kappa-light-chain-enhancer of activated B cells; NHS, N-hydroxysuccinimide; NLRP3, NOD-like receptor family pyrin domain-containing 3; OCN, osteocalcin; ONJ, osteonecrosis of the jaw; OPG, osteoprotegerin; OPN, osteopontin; PAb, Agaricus blazei polysaccharide; PBM, photobiomodulation; PDCD4, programmed cell death protein 4; PDGF, platelet-derived growth factor; PDGF-BB, platelet-derived growth factor-BB; PDL, periodontal ligament; PEG, polyethylene glycol; PI3K/AKT, phosphoinositide 3-kinase/protein kinase B; PRF, platelet-rich fibrin; PRP, platelet-rich plasma; PTH, parathyroid hormone; PTHLH, parathyroid hormone-like hormone; RAGE, receptor for advanced glycation end products; RANKL, receptor activator of nuclear factor κB ligand; rhBMP-2, recombinant human bone morphogenetic protein 2; ROS, reactive oxygen species; RUNX2, runt-related transcription factor 2; SDF-1, stromal cell-derived factor 1; sEV-AT, small extracellular vesicle derived from adipose tissue; siRNA, small interfering RNA; SP7, Sp7 transcription factor (osterix); SPP1, secreted phosphoprotein 1; TDN, tetrahedral DNA nanostructure; tFNA, tetrahedral framework nucleic acid; TGF-β, transforming growth factor beta; TLR4, Toll-like receptor 4; TNF-α, tumor necrosis factor alpha; β-TCP, beta-tricalcium phosphate; VEGF, vascular endothelial growth factor; VEGFA, vascular endothelial growth factor A; ZA, zoledronic acid; ZDPR, zoledronate/rapamycin-loaded nanoparticle.
